# Multiparametric Magnetic Resonance Imaging for Prediction of Parenchymal Hemorrhage in Acute Ischemic Stroke After Reperfusion Therapy

**DOI:** 10.1161/STROKEAHA.116.014343

**Published:** 2017-02-22

**Authors:** Kambiz Nael, James R. Knitter, Reza Jahan, Jeffery Gornbein, Zahra Ajani, Lei Feng, Brett C. Meyer, Lee H. Schwamm, Albert J. Yoo, Randolph S. Marshall, Philip M. Meyers, Dileep R. Yavagal, Max Wintermark, David S. Liebeskind, Judy Guzy, Sidney Starkman, Jeffrey L. Saver, Chelsea S. Kidwell

**Affiliations:** From the Department of Radiology, Icahn School of Medicine at Mount Sinai, New York, NY (K.N.); the Departments of Neurology and Radiology, University of Arizona, Tucson (J.R.K., C.S.K.); the Departments of Radiology and Neurosurgery (R.J.), Biomathematics (J. Gornbein), Neurology (D.S.L., J.L.S.), and Emergency Medicine and Neurology (J. Guzy, S.S.), University of California, Los Angeles; the Departments of Neurology (Z.A.) and Radiology (L.F.), Kaiser Permanente, Los Angeles, CA; the Departments of Neurosciences and the Stroke Center University of California, San Diego (B.C.M.); the Department of Neurology, Harvard Medical School, Massachusetts General Hospital, Boston (L.H.S.); Texas Stroke Institute, Dallas (A.J.Y.); the Departments of Neurology (R.S.M.) and Neurological Surgery and Radiology (P.M.M.), Columbia University College of Physicians and Surgeons, New York, NY; the Departments of Neurology and Neurosurgery, University of Miami, Jackson Memorial Hospital, FL (D.R.Y.); and the Departments of Radiology and Neurology Stanford University, CA (M.W.).

**Keywords:** biomarkers, embolectomy, hemorrhage, permeability, stroke

## Abstract

**Background and Purpose—:**

Patients with acute ischemic stroke are at increased risk of developing parenchymal hemorrhage (PH), particularly in the setting of reperfusion therapies. We have developed a predictive model to examine the risk of PH using combined magnetic resonance perfusion and diffusion parameters, including cerebral blood volume (CBV), apparent diffusion coefficient, and microvascular permeability (K2).

**Methods—:**

Voxel-based values of CBV, K2, and apparent diffusion coefficient from the ischemic core were obtained using pretreatment magnetic resonance imaging data from patients enrolled in the MR RESCUE clinical trial (Mechanical Retrieval and Recanalization of Stroke Clots Using Embolectomy). The associations between PH and extreme values of imaging parameters were assessed in univariate and multivariate analyses. Receiver-operating characteristic curve analysis was performed to determine the optimal parameter(s) and threshold for predicting PH.

**Results—:**

In 83 patients included in this analysis, 20 developed PH. Univariate analysis showed significantly lower 10th percentile CBV and 10th percentile apparent diffusion coefficient values and significantly higher 90th percentile K2 values within the infarction core of patients with PH. Using classification tree analysis, the 10th percentile CBV at threshold of 0.47 and 90th percentile K2 at threshold of 0.28 resulted in overall predictive accuracy of 88.7%, sensitivity of 90.0%, and specificity of 87.3%, which was superior to any individual or combination of other classifiers.

**Conclusions—:**

Our results suggest that combined 10th percentile CBV and 90th percentile K2 is an independent predictor of PH in patients with acute ischemic stroke with diagnostic accuracy superior to individual classifiers alone. This approach may allow risk stratification for patients undergoing reperfusion therapies.

**Clinical Trial Registration—:**

URL: https://www.clinicaltrials.gov. Unique identifier: NCT00389467.

Hemorrhagic transformation is a potentially devastating complication of reperfusion therapies in patients with acute ischemic stroke (AIS), in particular in the form of parenchymal hemorrhage (PH) that can result in poor outcome.^1^ Advanced imaging has been used for prediction of hemorrhagic transformation in patients with AIS with some success in previous studies. Several imaging features that have been associated with higher risk of developing PH include low apparent diffusion coefficient (ADC) values,^2,3^ increased microvascular permeability (K2),^4–6^ or a low cerebral blood volume (CBV).^7–10^

Although these individual imaging biomarkers have been associated with higher risk of PH, they have been analyzed individually rather than in combination. The purpose of this study was to identify whether multiparametric magnetic resonance imaging (MRI) using combined information from both MR diffusion and perfusion parameters can improve the overall diagnostic accuracy for prediction of PH using imaging data from the MR RESCUE trial (Mechanical Retrieval and Recanalization of Stroke Clots Using Embolectomy). In particular, we aimed to construct a predictive imaging model combining ADC, K2, and CBV, as an independent predictor of PH development.

## Methods

### Patients

The MR RESCUE trial was a phase 2b, randomized, controlled, multicenter trial conducted at 22 study sites in North America.^11^ Patients between the ages of 18 and 85 years, with National Institutes of Health Stroke Scale (NIHSS) scores of 6 to 29, who had a large-vessel, anterior-circulation ischemic stroke were randomly assigned within 8 hours after the onset of symptoms to undergo either mechanical thrombectomy

(Merci Retriever or Penumbra System) or standard medical care. All patients underwent pretreatment multimodal computed tomographic imaging or MRI of the brain and had follow-up imaging on day 7.

For this study, we included only patients who had initial diagnostic multimodal MRI including MR diffusion and perfusion as part of their evaluation.

### Analysis

#### Data Obtained From MR RESCUE Trial

Baseline and clinical data including patients’ age, sex, history of hypertension and diabetes mellitus, baseline systolic blood pressure, baseline NIHSS score were available from the MR RESCUE data set. Treatment type, if any, including IV tPA (tissue-type plasminogen activator) and mechanical thrombectomy were noted in addition to time to groin puncture in the subset of embolectomy patients when available. Patients with modified Rankin scale scores of 0 to 2 were classified as having a good functional outcome.

In the subset of patients who had catheter angiography for thrombectomy, primary revascularization was assessed with the use of the thrombolysis in cerebral infarction scale.^12^ Data were dichotomized using thrombolysis in cerebral infarction ≥2b as an indication of successful revascularization. Final revascularization status was assessed on day 7 computed tomography angiography or magnetic resonance angiography, and again data were dichotomized using thrombolysis in cerebral infarction ≥2b as a measure of successful revascularization. Successful global reperfusion was defined as a reduction of ≥90% in the volume of the perfusion lesion from baseline using time to maximum ≥6 seconds maps. Volume of infarction was calculated on pretreatment ADC maps using a threshold method (ADC <600×10^−6^ mm^2^/s). Favorable penumbral pattern on pretreatment MRI scans was defined per criteria used in the original study.^13^ Volume of Tmax ≥6 seconds was also assessed on baseline MR perfusion maps. The presence of PH was assessed on day 7 computed tomographic imaging or MRI (using gradient recalled echo images).

PH type 1 was defined as a hematoma occupying ≤30% of the infarcted territory with mild space-occupying effect; PH type 2 was defined as a hematoma encompassing >30% of the infarcted territory with substantial space-occupying effect or if hematoma occurred outside the infarcted area.^14^

#### Additional Image Analysis

Dynamic susceptibility contrast perfusion was processed using Food and Drug Administration–approved software (Olea Sphere 2.2; Olea Medical SAS, La Ciotat, France) applying a Bayesian probabilistic method.^15^ During dynamic susceptibility contrast acquisition, which is a T2*-weighted technique, after Gadolinium-contrast administration, there is typically an initial peak with the first pass of the bolus, often a smaller peak because of recirculation, followed by a constant decline to the baseline for the remainder of the acquisition, which is often 45 to 60 seconds. In the setting of blood–brain barrier disruption with increased microvascular permeability as Gadolinium crosses the blood–brain barrier, the concentration curve in an affected voxel reaches the baseline faster in comparison to normal brain tissue. This relative change compared with normal brain tissue can be molded to derive a measure of permeability referred to as K2.^16^

**Figure 1.**
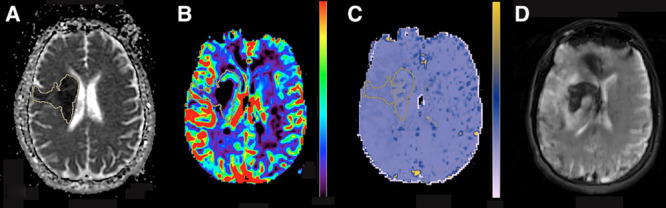
A 45-y-old man presented with left sided weakness and baseline National Institutes of Health Stroke Scale score of 19. Magnetic resonance imaging (MRI) was obtained 5.5 hours from symptoms’ onset presentation. Axial apparent diffusion coefficient (ADC; **A**), cerebral blood volume (CBV; **B**), and microvascular permeability (K2; **C**) from the pretreatment MRI are shown. Volume of interest (VOI) from the diffusion-weighted image hyperintense region was automatically generated, and after image coregistration, voxel values from VOI were used to calculate 10th percentile ADC (384×10^−6^ mm^2^/s), 10th percentile CBV (0.40), and 90th percentile K2 (0.32). Subsequent MRI 8 d later shows development of parenchymal hemorrhage in the region of infarction (**D**).

Dynamic susceptibility contrast maps were normalized to a region of interest placed in normal appearing white matter in the contralateral centrum semiovale. K2 was measured as percentage change in comparison to contralateral side and recorded in decimal unit. CBV was measured as ratios divided by normal contralateral value. Dynamic susceptibility contrast–derived CBV, K2, and ADC maps were coregistered with diffusion-weighted images for each patient using a 6-degree-of-freedom transformation and a mutual information cost function. Subsequently, a volume of interest from the diffusion-weighted image hyperintense region was calculated using a voxel-based signal intensity method subsuming the entire region of diffusion-weighted image hyperintensity (Figure 1). Voxel values from each volume of interest were used to calculate 10th percentile as the lowest representation of CBV and ADC and 90th percentile as the highest representation for K2 values in each patient.

### Statistical Analysis

Baseline characteristics including neuroimaging variables were compared between subjects with and without PH using *t* tests, χ^2^ tests, and Wilcoxon rank-sum tests as appropriate.

A multivariable regression model to predict PH was developed considering 6 imaging variables (10th percentile CBV, 10th percentile ADC, 90th percentile K2, ADC volume <600×10^−6^ mm^2^/s, favorable penumbral pattern, Tmax volume ≥6 seconds) and 8 clinical variables (age, sex, history of hypertension and diabetes mellitus, baseline systolic blood pressure, baseline NIHSS score, IV tPA administration, and mechanical thrombectomy). These imaging and clinical variables were entered into a backward (stepdown) logistic regression analysis, where variables were chosen from the set of candidates by backward elimination. A stepwise method was used to avoid collinearity because redundant variables were omitted. We also used a classification tree using the binary recursive partition model,^17^ which creates an upside-down tree based on binary splitting choosing a variable value that best separates those with PH from those without PH. The splits were chosen such that the groups formed were as different as possible. Accuracy statistics were computed to assess model performance including sensitivity, specificity, overall accuracy defined as (sensitivity+specificity)/2, and the area under the receiver-operating characteristic curve. Spearman correlations (*r*_s_) were computed, and scatter plots were examined to assess the association between 10th percentile CBV, 10th percentile ADC, and 90th percentile K2. Significance level was set at *P*=0.05 in our statistical analysis.

## Results

A total of 118 patients were enrolled in the MR RESCUE study. Of these, 83 patients had adequate baseline and follow-up MRI data to be included in our study. In 25 patients, multimodal computed tomography was performed as the baseline imaging modality. Ten patients were excluded because of inadequate and nondiagnostic MR perfusion data that did not meet our analysis criteria. Of those patients included in the analysis, 45 were men, 38 women, mean age was 66±15.2 years, and median and interquartile range of NIHSS score was 17 and 13 to 21. PH occurred in 20 out of 83 patients (24%; PH type 1 [n=14] and PH type 2 [n=6]).

The median and range of PH volumes were 16.5 mL and 9.4 to 80 mL. In 18 patients, PH occurred only within the infarction territory. In 2 patients, PH was seen within the infarction and remote from the infarction territory.

Demographic data and basic clinical information for patients with PH versus no PH are provided in Table 1.

**Table 1.**
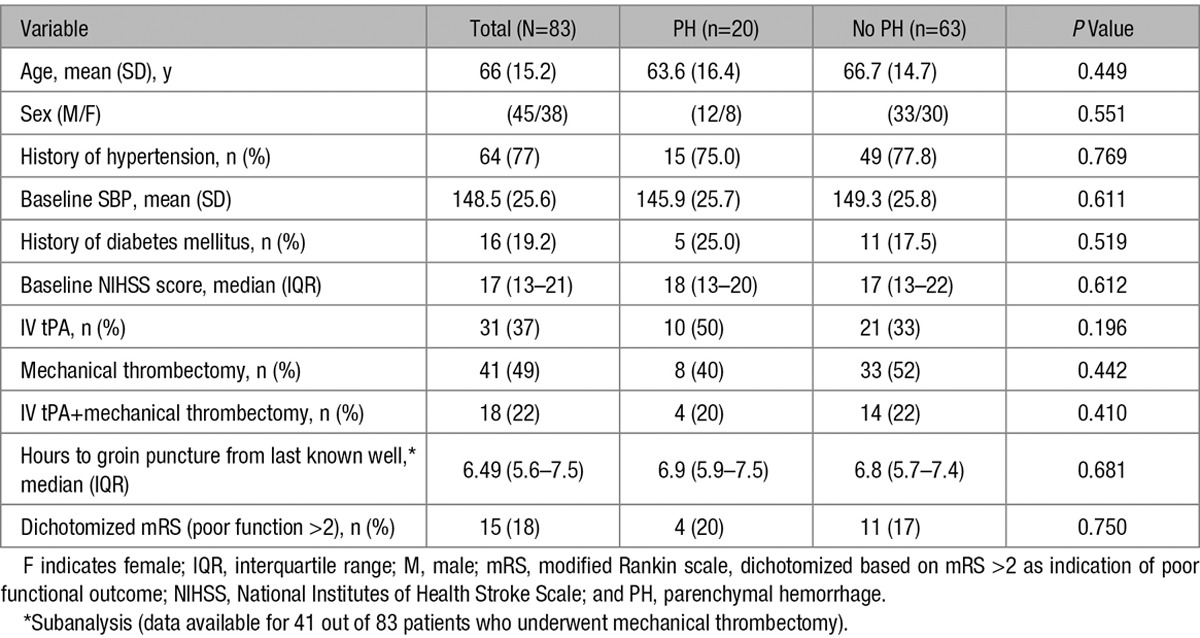
Baseline and Clinical Data in Patients With and Without PH

Among the 83 patients included in our analysis, a total of 31 received IV tPA and 41 received mechanical thrombectomy. Eighteen patients received both IV tPA and mechanical thrombectomy. IV tPA and mechanical thrombectomy were, respectively, applied in 50% and 40% of patients who developed PH, compared with 33% and 52% of patients without PH, and neither were significant contributor to development of PH (Table 1).

There was no significant difference in patients’ outcome between patients with and without PH when data were correlated with adjusted 90-day modified Rankin scale scores, using modified Rankin scale score >2 as indication of poor outcome.

### Imaging Data

**Figure 2.**
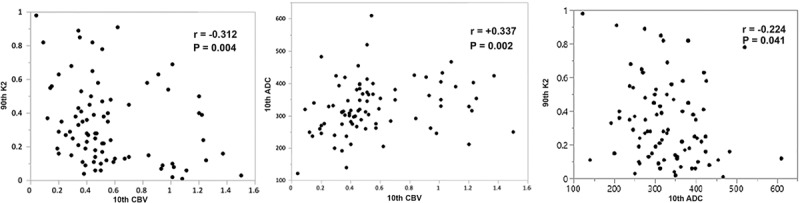
Scatter plots for 10th percentile cerebral blood volume (CBV), 10th percentile apparent diffusion coefficient (ADC), and 90th percentile microvascular permeability (K2). There are moderate negative correlations between 90th percentile K2 with 10th percentile CBV and 10th percentile ADC and a moderate positive correlation between 10th percentile CBV and 10th percentile ADC.

From the imaging data, volume of infarction, favorable penumbral pattern, baseline volume of Tmax ≥6 seconds, immediate postprocedural recanalization (subset analysis, only in 41 patients), D7 recanalization (subset analysis, day 7 vascular imaging available in 73 patients), and D7 global reperfusion (subset analysis, day 7 perfusion data only available in 62 patients) did not differ significantly between the 2 groups (Table 2). Patients with PH had significantly lower 10th percentile CBV and 10th percentile ADC values and significantly higher 90th percentile K2 values in comparison to patients without PH (Table 2). There was modest degree of correlation between these 3 imaging variables (Figure 2).

**Table 2.**
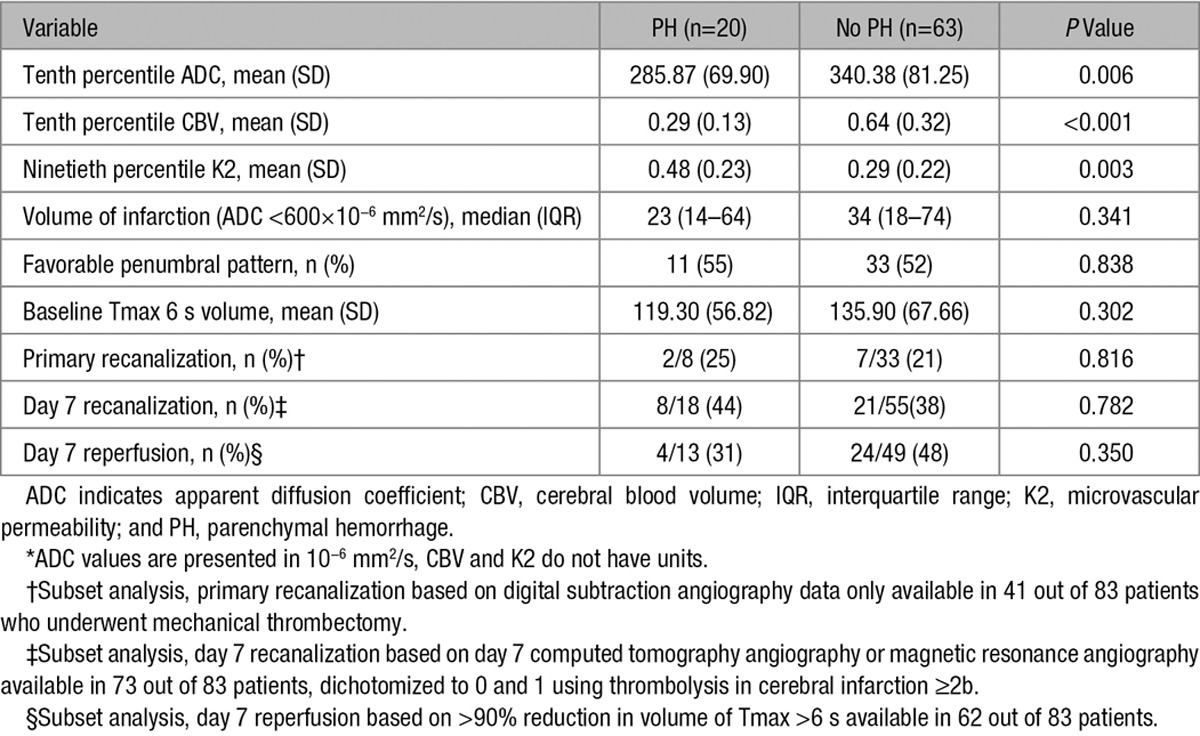
Imaging Data in Patients With and Without PH^*^

### Diagnostic Accuracy

The overall accuracy, sensitivity, specificity, and optimal threshold values for 10th percentile CBV, 10th percentile ADC, and 90th percentile K2 are shown in Table 3.

**Table 3.**
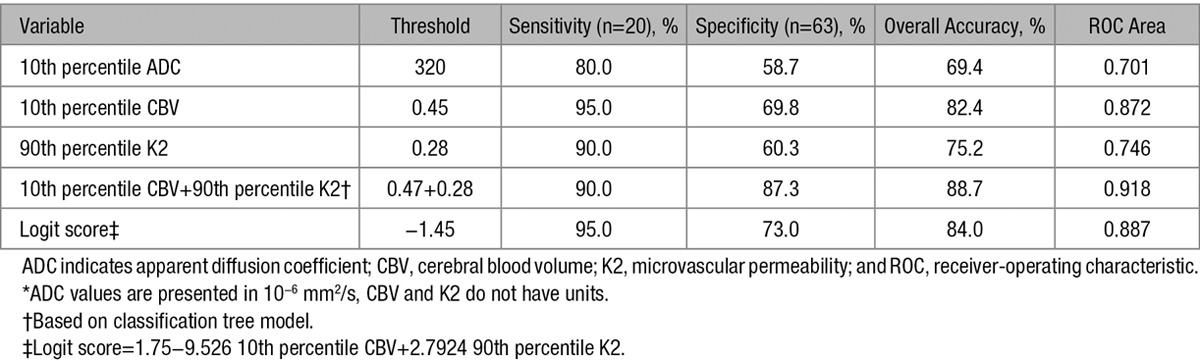
Optimal Threshold, Sensitivity, Specificity, and Overall Accuracy for 10th Percentile CBV, 10th Percentile ADC, and 90th Percentile K2^*^

Using backward logistic regression model, only 2 variables (10th percentile CBV and 90th percentile K2) were found to be simultaneously significant and remained independent predictor of PH when evaluated against other clinical and imaging parameters.

**Figure 3.**
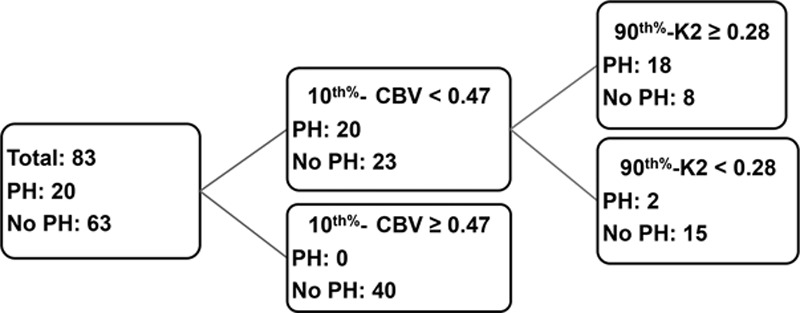
Classification tree model using combined 10th percentile cerebral blood volume (CBV) and 90th percentile microvascular permeability (K2). If 10th percentile CBV ≥0.47 and 90th percentile K2 <0.28, the model predicts no parenchymal hemorrhage (PH) in 55 out of 63 patients. If 10th percentile CBV <0.47 and 90th percentile K2 ≥0.28, the model predicts PH in 18 out of 20 patients.

The score given by the logistic model was logit score=1.75 to 9.526 10th percentile CBV+2.7924 90th percentile K2. This score with a threshold of −1.45 correctly classified 19 out of 20 PH (95% sensitivity) and 46 out of 63 no PH (73.0% specificity) corresponding to an overall accuracy of 84.0%. The receiver-operating characteristic curve area was 0.887. Subsequently, our classification tree model using a binary recursive partition method also confirmed significant contributions from the same 2 variables (10th percentile CBV and 90th percentile K2), resulting in improvement of overall diagnostic accuracy superior to each measure alone (Table 3). Using 10th percentile CBV at a threshold of 0.47 and 90th percentile K2 at a threshold of 0.28, we were able to identify 18 out of 20 patients with PH (90.0%) and 55 out of 63 patients without PH (87.3%) correctly (Figure 3) for an overall accuracy of 88.7%. The receiver-operating characteristic area was 0.918.

## Discussion

In patients with AIS, development of hemorrhagic transformation, particularly PH, is associated with poorer prognosis,^1^ and its prediction may have critical therapeutic implications when considering reperfusion therapies.^18^ This analysis from the MR RESCUE data set demonstrates that multiparametric MRI, using routinely obtained MR diffusion and perfusion biomarkers, can be used for prediction of PH in patients with AIS with high diagnostic accuracy.

We would like to highlight 2 significant findings from this study. First, AIS patients who had developed PH had lower baseline ADC and CBV values and higher baseline K2 values in the ischemic region in comparison to patients without PH. For CBV, we showed that having low CBV in the infarction lesion was the strongest single classifier to predict development of PH in accordance with several other reports.^7–10^ In our cohort, a 10th percentile CBV as the minimum representation of CBV in the diffusion-weighted images hyperintense region at threshold of 0.45 predicted development of PH with a sensitivity of 95% and specificity of 69.8%, which is concordant with recently reported results by Mishra et al,^7^ who reported a sensitivity of 94% and specificity of 63%, using a threshold of 0.42.

With regard to permeability, we showed that increased K2 within the infarction lesion is associated with increased risk of PH in patients with AIS, a finding that is also in accordance with previous reports.^4,5^ In our study, a threshold of 0.28 using 90th percentile K2 as the highest representation of permeability predicted development of PH with a sensitivity of 90% and specificity of 60.3%. We also showed that AIS patients who developed PH had significantly lower baseline ADC values within the infarction lesion as shown previously.^2,3^ In our study, using 10th percentile ADC at threshold of 320×10^−6^ mm^2^/s resulted in prediction of PH with sensitivity of 80% and specificity of 58.7%.

Previous MRI studies have shown that ADC or perfusion values such as CBV within acute infarction lesions are heterogeneous, and therefore, averaging the values of the entire lesion does not accurately discriminate the inherent regional variations.^19,20^ Use of minimum or maximum values obtained from averaged voxel values does not represent the lowest or highest representation of a particular classifier across different ischemic regions. In this study, we opted to use a unique approach of applying voxel-based analysis of the entire infarction lesion using 10th percentile (for ADC and CBV) and 90th percentile (for K2) values to represent the lowest and highest representation of each classifier, rather than using mean values to avoid this potential confounding factor.

We think that the observed imaging findings represent the underlying pathophysiology and functional status of the ischemic tissue, which can lead to an increased risk of PH development. Low ADC values have been correlated with greater degrees of ischemia,^21^ which in turn increase the risk of secondary hemorrhage. In addition, severe brain hypoperfusion, evident by low CBV, is a hallmark of imminent neural and tissue death.^22^ Severe ischemia and critical hypoperfusion, in turn, can result in severe damage to the microvasculature and disruption of the blood–brain barrier (evident by increase in K2). This combination (low ADC as a marker of tissue ischemia, low CBV as a marker of degree of ischemia, and high K2 as a marker of increased permeability) can in turn increase the risk of hemorrhage, particularly in the setting of reperfusion.^23,24^

Our second significant finding is that combined K2 and CBV remained as an independent classifier to predict hemorrhage and improved the predictive power of our imaging model compared with other classifiers individually or in combination. Using a combination of 10th percentile CBV at threshold of 0.47 and 90th percentile K2 at threshold of 0.28 resulted in prediction of PH with overall sensitivity and specificity of 90% and 87%, respectively. The major strength of this model is a substantial increase in overall specificity to 87% (for combined 10th percentile CBV and 90th percentile K2) in comparison to 69.8% (for 10th percentile CBV) and 60.3% (for 90th percentile K2) when used alone for detection of PH.

This translates to a decrease in number of false positives from 25 for 90th percentile K2 to 19 for 10th percentile CBV to 8 for combined 10^th^ percentile CBV and 90th percentile K2. Given these test characteristics with high specificity, the absence of low CBV (10th percentile CBV >0.47) and absence of high permeability (90th percentile K2 <0.28) can be used to reassure physicians and patients with a low risk of PH after thrombolytic treatment. Having high specificity for a predictive test is of paramount importance and can prevent under-treatment patients with AIS in fear of PH development.

The impact of nonimaging variables on risk of PH remains unclear in patients with AIS. In our study, none of the baseline and clinical data, such as age, baseline NIHSS score, volume of infarction, and history of hypertension or diabetes mellitus were associated with increased risk of PH as shown by others.^8,25^ This may be because of the fact that the imaging variables are mediators in the association of some of these variables or because of unique characteristics of the MR RESCUE sample. Similarly, the impact of reperfusion therapies on risk of hemorrhagic transformation requires further study. Some studies reported significant association with reperfusion,^7,25^ whereas others, like ours, did not.^8,9^ In our study, the reperfusion and revascularization status were not significantly associated with development of PH as shown by others.^8^ In particular, 10th percentile CBV, 90th percentile K2, and combined 10th percentile CBV and 90th percentile K2 remained independent predictor of PH development, regardless of whether global reperfusion and recanalization were achieved. It should be noted that only a subset of our patients (49%) underwent mechanical thrombectomy, and therefore, lack of association between early revascularization status and PH development should be interpreted in this context and particular cohort. Moreover, revascularization rates within this subset were substantially lower than that in other studies, including recent trials of stent retriever devices. Recent studies have shown early revascularization or early reperfusion; in particular, reperfusion of a lesion with low CBV may promote and increase risk of PH development.^7,25^ This analysis could not be reproduced in our study because of absence of early reperfusion data. The MR RESCUE study was not designed to assess the effect of early reperfusion, and follow-up imaging was not performed until day 7.

There are several limitations to our study. The small number of PHs may limit the statistical power of our results. This study is a post hoc analysis of a clinical trial data set of a modest size, and therefore, the predictive power of our imaging model needs to be validated in an independent prospective and larger cohort before it can be applied to general practice. Although all PH developed before the follow-up imaging, the exact time of PH development was not available from our retrospective cohort. We were only able to analyze PH (rather than symptomatic intracranial hemorrhage because of the overall small number of patients in MR RESCUE with symptomatic intracranial hemorrhage). Lack of association between poor outcome and development of PH should be interpreted with caution because only 6 patients had symptomatic PH. It should be noted that the relationship between radiological type of hemorrhage and symptomatology remains largely uncertain. Symptomatology of a PH may be a function of lesion location and degree or volume of hemorrhage, rather than differences in pathophysiology.^23^

## Conclusions

In the MR RESCUE data set, using multiparametric MRI, we found that combined 10th percentile CBV and 90^th^ percentile K2 independently predicted development of PH in patients with AIS with a diagnostic accuracy superior to individual classifiers alone. Severe reduction of CBV and increased permeability within the infarction bed identified patients at risk of PH regardless of type of thrombolysis and recanalization status. If its potential is realized in a larger independent prospective cohort, this combined classifier may be used to optimize or guide stroke treatment decision making.

## Sources of Funding

Financial support was received from the National Institutes of Health/National Institute of Neurological Disorders and Stroke P50 NS044378 for all authors except Dr Nael and JR Knitter.

## Disclosures

Dr Saver has potential conflicts of interest with Stryker, Medtronic, Neuravia, Boehringer Ingelheim, and Genentech, Dr Yoo has potential conflicts with Penumbra and Neuravi, Dr Schwamm has potential conflicts with Genentech, Lundbeck, and Penumbra, Dr Meyer has potential conflicts with Genentech, Dr Jahan has potential conflict with Medtronic Neurovascular, Dr Nael has potential conflict with Olea Medical, and Dr Yavagal has potential conflict with Medtronic. The other authors report no conflicts.
